# Research on the ring formation mechanism of magnesian flux pellets in rotary kiln

**DOI:** 10.1038/s41598-023-29440-z

**Published:** 2023-02-10

**Authors:** Zongheng Guo, Tielei Tian, Yuzhu Zhang

**Affiliations:** College of Metallurgy and Energy, North China University of Science and Technology, Tangshan City, Hebei Province China

**Keywords:** Engineering, Chemical engineering

## Abstract

The forming of rings was a key problem of magnesian flux pellets in rotary kiln, which seriously limited production efficiency. Pellet powder and flux were the raw materials of the ring. Based on this, the bonding strength, melting behavior and microstructure of pellet powder and its mixed powder with flux were investigated. The influence of basicity (R = CaO/SiO_2_) on ring behavior of pellet powder was analyzed, and ring formation mechanism of magnesian flux pellets was clarified. The results showed that acid pellet powder was not easy to form rings due to lower bonding strength of briquettes. Due to changes in bonding process after mixing of flux, magnesian flux pellet powder produced ferrite and silicate liquid phase with lower melting point, which promoted diffusion and recrystallization of hematite and enhanced compressive strength of briquettes, then finally caused rings to form. Moreover, it is required to control roasting temperature below 1200 °C, which is a necessary condition for magnesian flux pellet powder to form an initial ring which was easy to be destroyed.

## Introduction

With the dual measures of vigorously cutting excessive industrial capacity and environmental renovation, China's steel industry was under the pressure of structural adjustment and upgrading^1^, which forced iron and steel enterprises to embark on the clean, efficient and high-quality development route. Magnesian flux pellet has become a high-quality and efficiency blast furnace raw material with high grade, low energy consumption and environmental protection^2–4^. According to statistics, compared with sintering process, pollutants CO_2_, SO_2_ and NO_x_ produced by tons of products in pelletizing process were reduced respectively by 75%, 53% and 16%, and energy consumption in pelletizing process was reduced by 11.9%^5,6^. Therefore, pelletizing process was more environmental friendly than sintering process.

Production processes of pellets mainly included shaft furnace, belt roaster and grate rotary kiln^7–9^. Grate rotary kiln was compatible with a variety of fuels for heating^10^. Moreover, China is rich in coal resources, with an output accounting for about 60% of total pellet production^11^. Grate rotary kiln process occupied a mainstream position in China's pellet production. However, rotary kiln process could easily form rings in production of pellets. Especially in production process of magnesian flux pellets, rings were formed frequently in a short cycle, which has seriously limited the process of industrial production of magnesian flux pellets.

At present, there are few reports on the growth behavior and formation mechanism of the ring formed by magnesian fluxed pellets in the rotary kiln, mainly focusing on the reaction between acid pellets, fluxed pellets and coal and coal ash in the rotary kiln^12–14^. Previous studies have shown that the ring of rotary kiln during the production of pellets from hematite mainly comes from preheated pellet powder and coal ash ^15,16^. Previous researchers have shown that it is difficult for pure pellet powder to form the ring due to insufficient Fe_2_O_3_ recrystallization in the rotary kiln, but coal ash can strengthen the bond strength, which make the initial ring formed by mixed powder difficult to be destroyed^17–19^. Sefidari et al. studied the influence of adding biomass into coal on the ring formation in rotary kiln, and established the relationship between the ring formation trend and ash melting viscosity^20^. The formation mechanism of ring at low temperature is mainly that unburned coal powder reduces hematite to FeO and reacts with coal ash to form silicate phase with low melting point, which produce liquid phase in low temperature and promote the adhesion of hematite particles; the formation mechanism of the ring at the high temperature is mainly the crystallization and diffusion of hematite, and the liquid phase plays a secondary role in the formation of the ring^17,20,21^.

However, due to the variability of components of magnesian fluxed pellets , the influence of magnesian fluxed pellets on the ring formation in rotary kiln has not been clearly defined. Therefore, it is very necessary to study the formation mechanism of magnesian fluxed pellets in rotary kiln. In this study, briquettes of magnesian fluxed pellet powder with different components were prepared and roasted to investigate their bonding strength. At the same time, the influence of different roasting temperature on the bonding strength of pellet powder was also studied. The microstructure, morphology and composition of the powder briquettes were observed by polarizing microscope, XRD, SEM and EDS. The chemical composition and liquid phase proportion in the powder briquettes was calculated by FactSage software^22^.

## Experimental materials and methods

### Preparation of pellet powder raw materials

The iron concentrate powder, flux and bentonite used to prepare pellets powder raw materials are from a Chinese iron and steel company, and their chemical composition is shown in Table 1. According to the actual production of the pellet plant, five kinds of pellets powder with different basicity (R = CaO/SiO_2_) are designed, with basicity of 0.6, 0.8, 1.0, 1.2 and 1.4 respectively, fixed SiO_2_ content of 5.0% and MgO content of 2.0%, as shown in Table 2. PMC mine, Yanshan mine and Miaogou mine are mixed according to the proportion in Table 2, then dolomite and limestone are added to adjust the basicity, MgO content and SiO_2_ content of mixed mineral powder, then 0.1% bentonite is added and fully mixed.

**Table 1:** Chemical compositions of experimental raw materials (wt%).

Raw materials	TFe	FeO	SiO_2_	CaO	MgO	Al_2_O_3_	Burnt loss
PMC mine	66.70	28.57	0.76	0.63	2.56	0.094	− 1.60
Yanshan mine	65.40	24.28	5.91	0.88	0.59	0.45	− 2.50
Miaogou mine	65.64	26.86	6.44	0.65	0.65	1.76	− 3.00
Dolomite	–	–	0.86	30.23	22.24	0.24	38.52
Limestone	–	–	4.02	51.36	0.46	1.22	42.58
Bentonite	–	–	55.96	4.16	2.01	11.73	–

**Table 2:** Composition of pellets with different basicity(wt%).

Basicity	Composition				
	PMC mine	Yanshan mine	Miaogou mine	Dolomite	Limestone
0.6	16.93	35.35	42.16	4.87	1.63
0.8	16.92	35.35	40.27	4.80	3.57
1.0	16.91	35.35	38.42	4.74	5.47
1.2	16.90	35.35	36.60	4.68	7.35
1.4	16.89	35.35	34.81	4.63	9.19

According to the production of the pellet plant, the mixed mineral powders with different basicity are prepared into pellets. The briquettes are calcined in a tubular furnace under the conditions of preheating temperature of 950 °C for 10 min, and roasting temperature of 1250 °C for 10min^23,24^. After cooling to room temperature, the briquettes are prepared into pellets powder of more than 200 meshes through a grinding machine, which is used to simulate and replace the magnesian fluxed pellets powder produced in rotary kiln. It can be seen from the standard sieve that 80% of the obtained powders have a particle size of more than 200 meshes, and 20% have a particle size of less than 200 meshes.

### Briquetting, preheating and roasting

The above pellets powder with different basicity are briquetted, and then 3 g pellets powder are prepared into briquettes by using a steel cylinder mold with an inner diameter of 10 mm under the pressure of 15Mpa with the cooperation of a hydraulic press^30^.Generally, the preheating temperature in rotary kiln is 950 °C for 10 min, and the roasting temperature is 1250 °C for 10 min, when rotary kiln producing pellets. The briquettes are preheated and roasting in a tubular furnace under preheating temperature of 950 °C for 10 min and roasting temperature of 1250 °C for 10 min^23,24^. After preheating and roasting, the briquettes were cooled to room temperature.

### Melting characteristic

A certain quality of pellets powder is mixed with dextrin aqueous solution, and a triangular cone is prepared according to a certain size. The triangular cone is put into a tubular furnace and heated at a certain rate in a soft reducing atmosphere. High temperature camera was used to observe the deformation of triangular cone. Four melting characteristic temperatures are recorded according to Chinese standard (GB/T 219–2008): deformation temperature, softening temperature, hemisphere temperature and flow temperature^25^.

### Experimental methods

The compressive strength is used to evaluate the bonding strength of the briquettes. The higher the compressive strength is, the easier the pellets powder is to form rings in rotary kiln. The compressive strength tester is used to test the compressive strength of the briquettes. When the briquettes are broken, the strength on the compressive strength meter is regarded as the compressive strength of the briquettes.Three briquettes were measured for each test and their average value was considered as the compressive strength. The deformation temperature, softening temperature, hemispherical temperature and flowing temperature of pellets powder with different basicity content were measured by melting point and melting rate meter. The lower the deformation temperature and softening temperature, the more the content of low melting point material in the pellet powder, and the more liquid phase produced in the powder at high temperature. The increase of liquid phase will enhance the bonding strength of the powder. The briquettes without cracks were selected for polishing, and then the mineral phase structure of the briquettes was analyzed by Quanta 650 field emission scanning electron microscope and DM4500P research grade polarizing microscope. D/MAX2500PC X-ray diffractometer was used to analyze the briquettes by XRD. SEM–EDS was used to analyze the microstructure and element distribution of the briquettes. The proportions of the liquid phase in the bonding phase was calculated by FactSage software^26^.

## Results and discussion

### Effect of basicity and roasting temperature on compressive strength of briquettes

**Figure 1:**
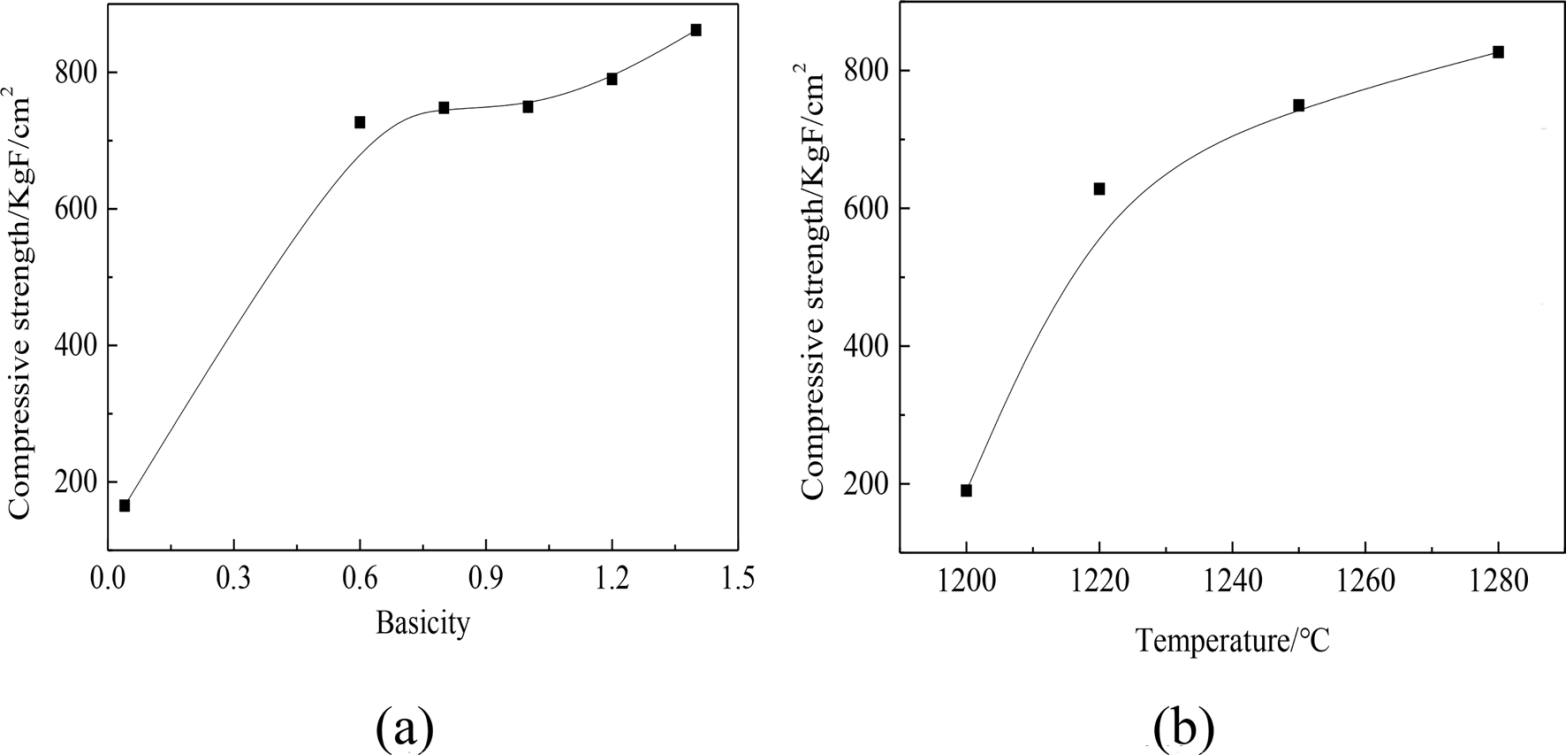
The effect of basicity and roasting temperature on compressive strength of briquettes.

Compressive strength were investigated on briquettes with basicity of 0.6, 0.8, 1.0, 1.2 and 1.4, SiO_2_ and MgO content of 5.0% and 2.0% respectively, which have been preheated, roasted and cooled.The experimental results are shown in Fig. 1a.

Figure 1a showed that there was a positive correlation between compressive strength of briquettes and basicity. The compressive strength of acid briquettes was 165.4KgF/cm^2^, which was lower than basicity of briquettes. When basicity was 0.6, compressive strength of briquettes was 726.8KgF/cm^2^. When basicity ranged from 0.6 to 1.0, compressive strength of briquettes changed little. When basicity exceeded 1.0, compressive strength of briquettes increased. When basicity was 1.4, compressive strength of briquettes was 861.8KgF/cm^2^.

There was a positive correlation between the compressive briquette strength and the basicity.According to the correlation analysis in mathematical statistics, the Pearson correlation coefficient(r, − 1 ~ 1) between basicity and compressive strength is 0.87743, indicating that there is a strong correlation between basicity and compressive strength. On the one hand, the flux changed the composition of pellet powder and improved the bonding process of pellet powder. On the other hand, the increase of basicity also leaded to the promotion of CaO content, so that the excess CaO entered the slag phase and formed a liquid phase with low melting point. These formed liquid phases would benefit the promotion of the hematite recrystallization and greatly enhanced the compressive strength of briquettes, which made the compressive strength changed greatly^24,27–29^.

Acid briquettes have a low compressive strength, so did rings formed in rotary kiln. Under the condition of original basicity. It was very easy to be destroyed and it was difficult to form initial rings, which made the ring formation cycle of acid pellets longer in the production process^30^. However, upon increase in basicity, pellet powder in rotary kiln was highly improved in compressive strength, and form unbreakable initial rings very easily. With an increase in pellet powder in rotary kiln, initial rings gradually aggravated, which hindered the movement and circulation of materials and hot gas flow, causing a reduction in magnesian fluxed pellets quality and production.

Furthermore, Fig. 1b showed that with a decrease in roasting temperature, the compressive strength of briquettes gradually decreased to 190.1KgF/m^2^ at 1200 °C, which was close to that of acid briquettes. Therefore, under the condition of ensuring pellet strength, roasting temperature should be controlled below 1200 °C, which is a necessary condition for production of magnesian flux pellets.

### The effect of basicity on melting temperature of briquettes

**Figure 2:**
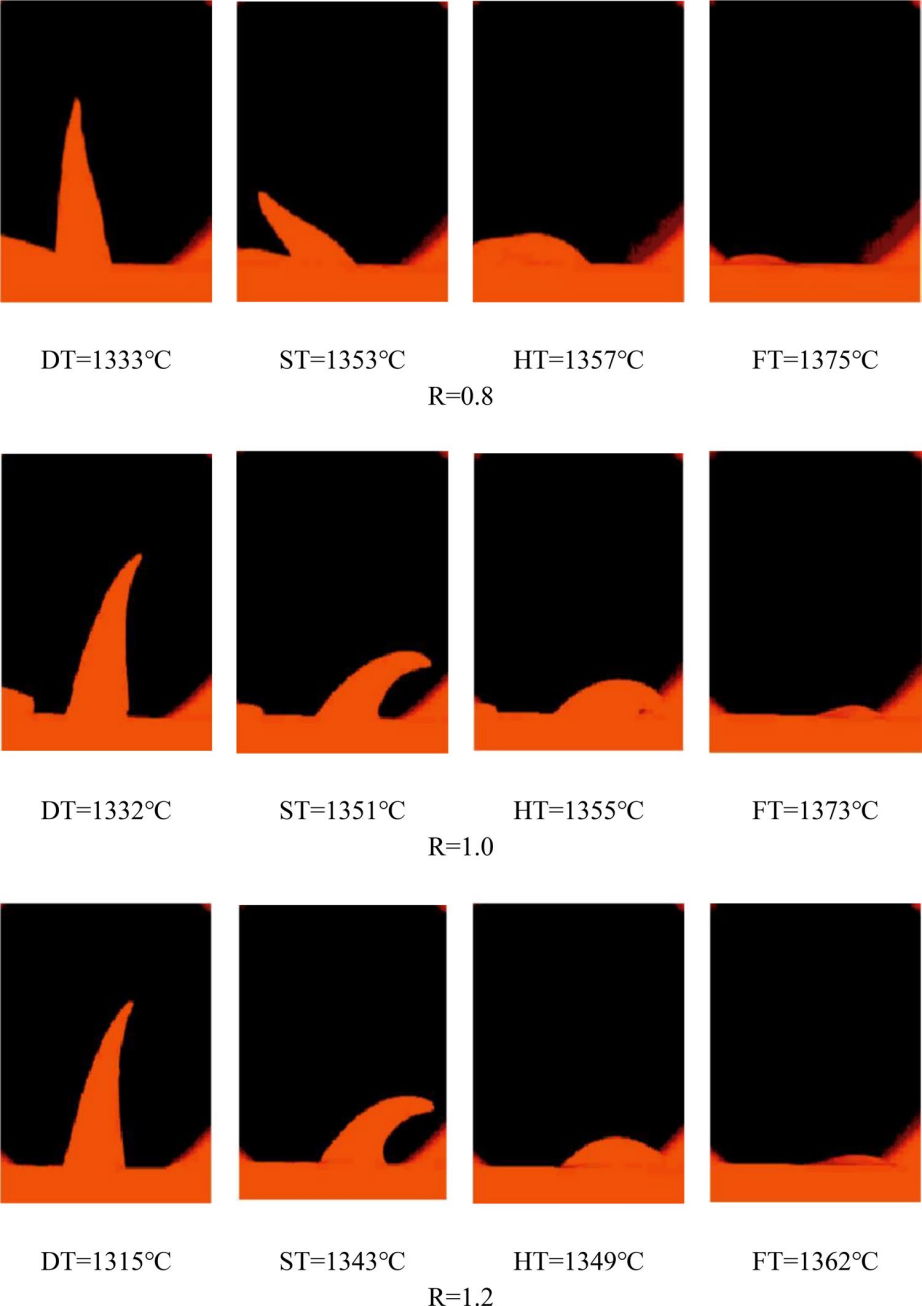
The effect of basicity on melting temperature of briquettes.

The melting characteristic temperature of pellets powder with basicity of 0.8, 1.0 and 1.2, SiO_2_ and MgO content of 5.0% and 2.0% respectively was measured by melting point melting rate meter. The experimental results are shown in Fig. 2.

As shown in Fig. 2, there was a negative correlation between the deformation temperature and softening temperature of briquettes and the basicity. According to the Fe_2_O_3_-CaO phase diagram^9^, when w(CaO) was less than 20%, liquidus temperature decreased gradually with an increase in CaO content. Therefore, the CaO content in briquettes would increase with the increase of basicity, which promoted the formation of low-melting-point mineral phase and increased the amount of liquid phase, leading to a lower deformation temperature and softening temperature of briquettes^20^.This means that with the increase of basicity, the deformation temperature of the briquettes decreases and the compressive strength of the briquettes increases.The formation of the ring of magnesian fluxed pellets in rotary kiln will be more serious.

### XRD and Mineral phase structure analysis

**Figure 3:**
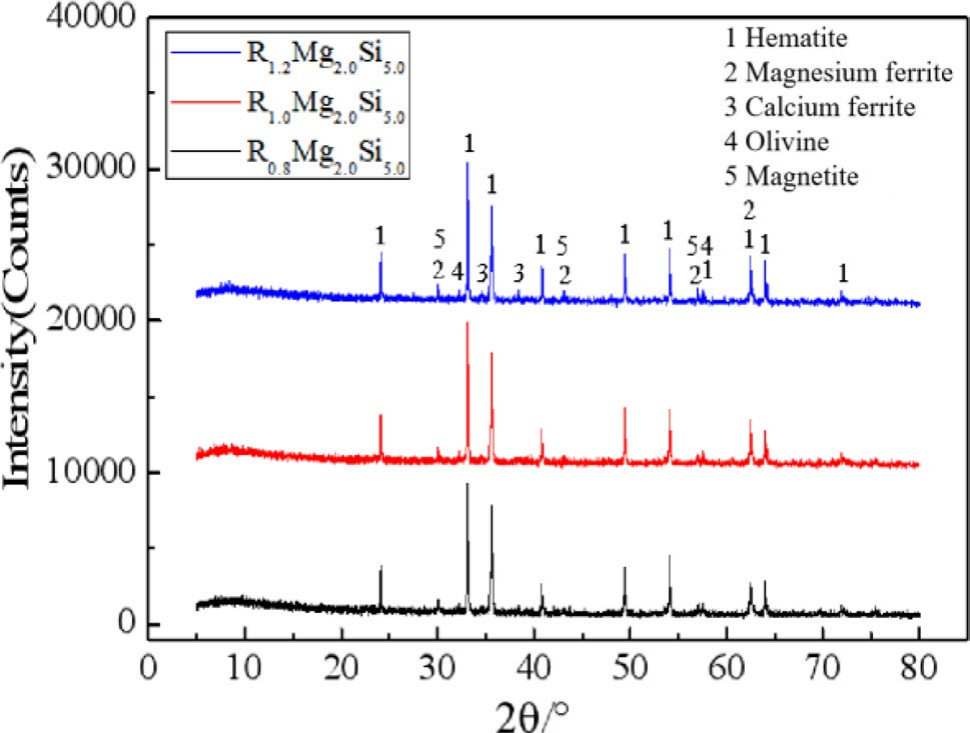
XRD diffraction results of beiquettes briquettes with different basicity.

Figure 3 showed that metal phase of briquettes was mainly composed of a large amount of hematite and a small amount of magnetite, while the bonding phase was mainly composed of silicate and calcium silicate and the melting point of olivine is 1205 °C, the melting point of magnesium ferrite is 1720 °C, and the melting point of calcium ferrite is 1226 °C^31^. With the increase of basicity, the ferrite phase and silicate phase gradually increased (as shown in formulas 1, 2 and 3), and the iron olivine phase gradually transmuted into the calcium iron olivine phase with a lower melting point, which increased the amount of liquid phase, accelerated the diffusion of hematites and filled the pores of the briquettes, leading to a larger compressive strength of briquettes^24,32^.

1 $$ {\text{Fe}}_{{2}} {\text{O}}_{{3}} \left( {\text{s}} \right) + {\text{CaO}}\left( {\text{s}} \right) \to {\text{CaO}} \cdot {\text{Fe}}_{{2}} {\text{O}}_{{3}} \left( {\text{l}} \right) $$

2 $$ {\text{FeO}}\left( {\text{s}} \right) + {\text{SiO}}_{{2}} \left( {\text{s}} \right) \to {\text{FeO}} \cdot {\text{SiO}}_{{2}} \left( {\text{l}} \right) $$

3 $$ {\text{CaO}}\left( {\text{s}} \right) + {\text{FeO}}\left( {\text{s}} \right) + {\text{SiO}}_{{2}} \left( {\text{s}} \right) \to {\text{CaO}} \cdot {\text{FeO}} \cdot {\text{SiO}}_{{2}} \left( {\text{l}} \right) $$

**Figure 4:**
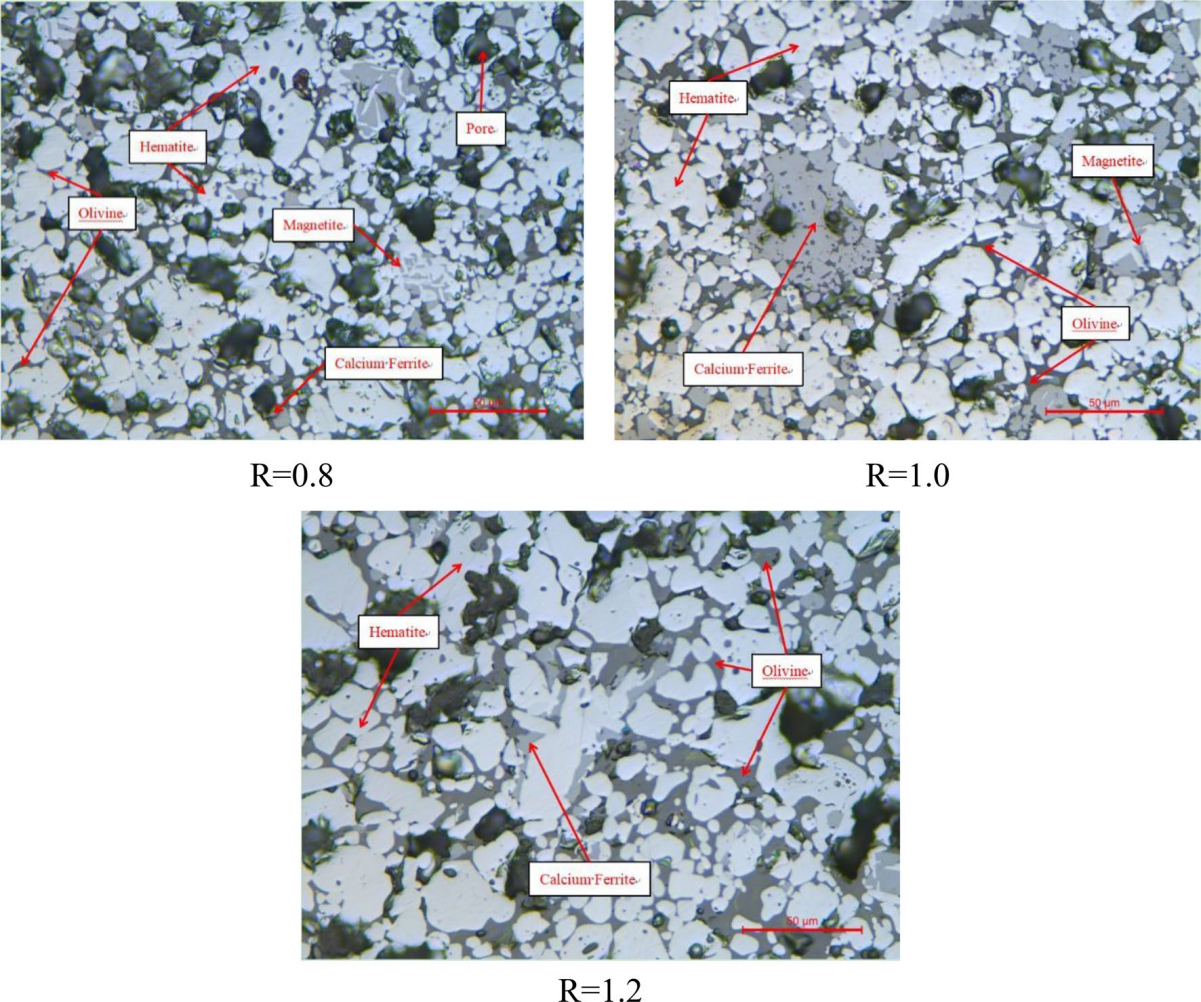
Mineral phase structure of briquettes with different basicity, reflected light(× 500).

Figure 4 showed a dense and uniform microstructure distribution of briquettes with different basicities and also a uniform distribution of pores which are of different sizes and irregular shapes. The ore phase was mainly composed of hematite and a small amount of magnetite, calcium ferrite and calcium iron olivine, which revealed the same results of XRD analysis.

With changes in basicity of briquettes, content of hematite changed little, which increased the content of calcium ferrite and calcium iron olivine. When basicity ranged from 0.8 to 1.0, hematite crystals were recrystallized and grown, which gradually formed slag phase consolidation with an increase of calcium ferrite, and the pores decreased due to the filling of iron calcium olivine^28^. When the basicity ranged from 1.0 to 1.2, there would be little changes in pores and a large area of continuous crystallization of hematites. Moreover, a large amount of iron calcium olivine and some calcium ferrite were intertwined between hematite crystals, which enhanced recrystallization of hematites, thus, increased the density of briquettes^33^.

To sum up, with the increase of basicity, the hematite recrystallized and grew while a large amount of iron calcium olivine and some calcium ferrite were intertwined between hematite crystals to enhanced recrystallization of hematite. This caused briquettes to have a smaller porosity, a higher density and a larger compressive strength, which made the rings in rotary kiln more dense and unbreakable.

### Elements distribution analysis

**Figure 5:**
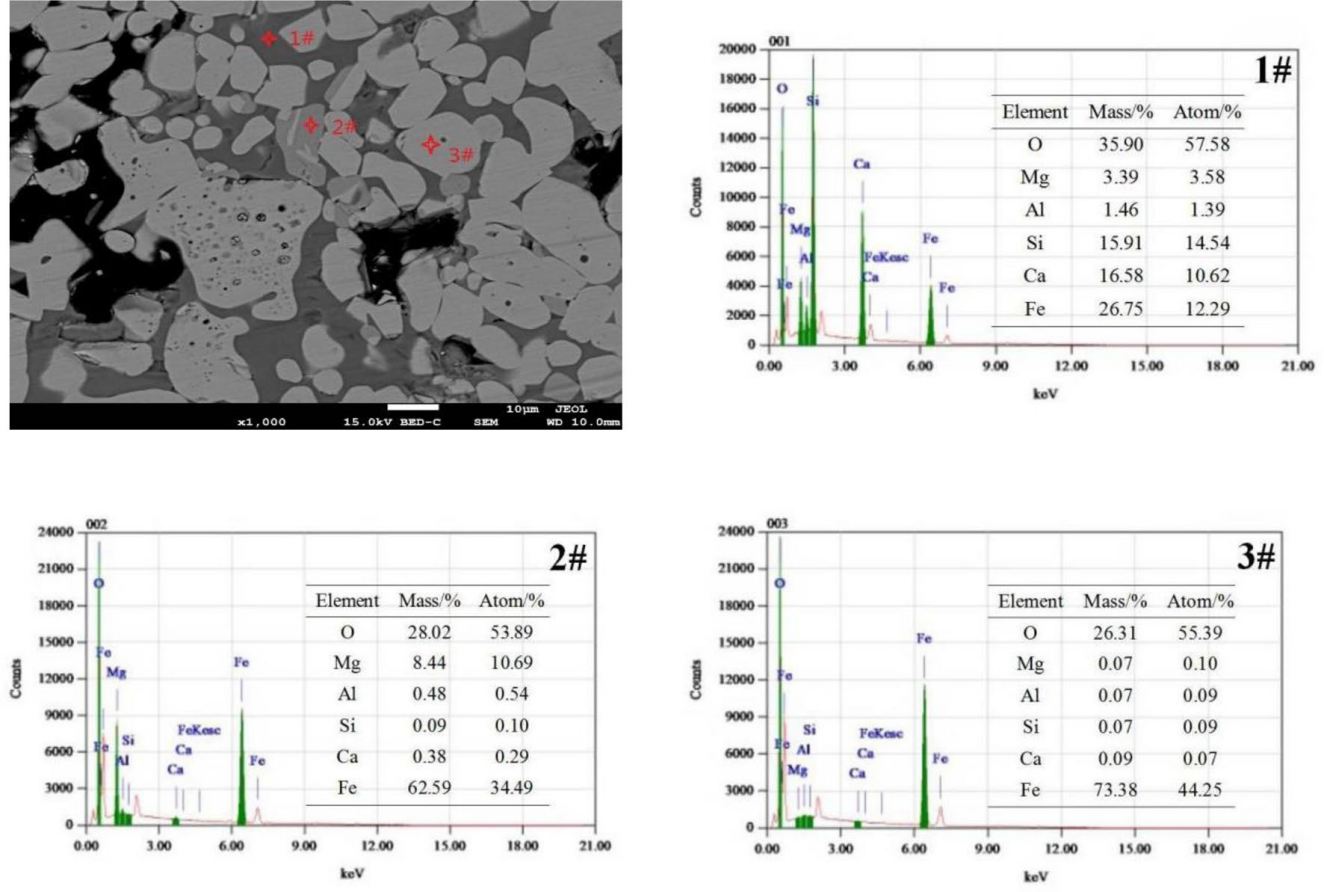
ESD analysis of briquettes with basicity 1.0, MgO 2.0% and SiO_2_ 5.0%.

As shown in Fig. 5, the element composition at 3# showed that this phase was hematite; the element composition at 2# showed that the phase was magnetite with a small amount of Mg, and Mg^2+^ limited the oxidation of magnetite in the magnetite lattice, which made magnetite amorphous and almost not distributed around hematite; the element composition at 1# showed that this phase was liquid phase which distributed around hematite, mainly due to the reaction of hematite with silicate phase to form a large number of ferrite liquid phase with low melting point, which accelerated the mass transfer of Fe^3+^, improved the recrystallization ability of hematite and enhanced the compressive strength of briquettes^34^. This also showed that higher basicity would make briquettes have a larger compressive strength and exacerbate the ring formation in the rotary kiln, which impeded the production of magnesian flux pellets.

### Phase diagram analysis

**Figure 6:**
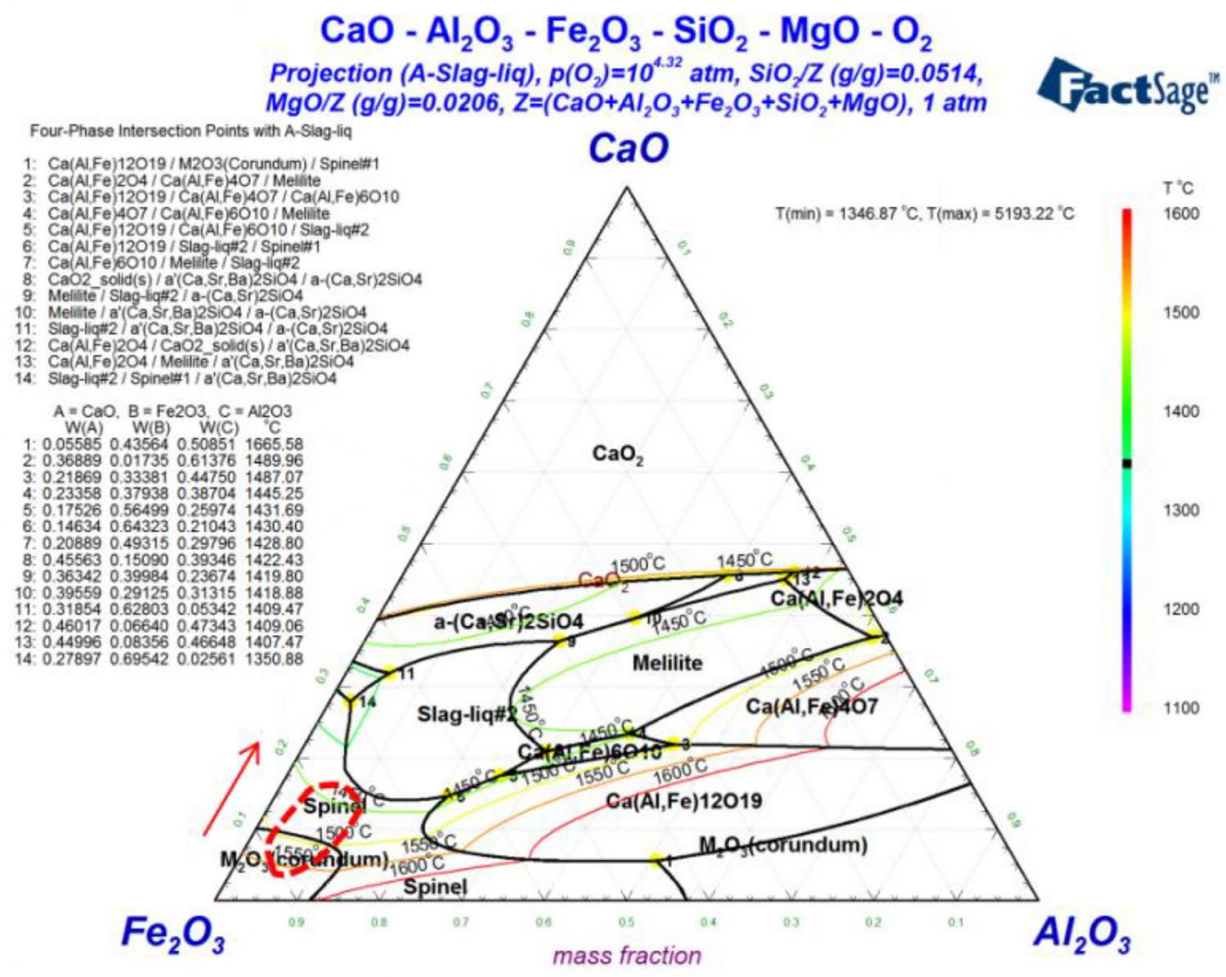
Phase diagram of CaO-Al_2_O_3_-Fe_2_O_3_-5wt%SiO_2_-2wt%MgO (calculated by FactSage 8.2).

Figure 6 shows the phase diagram of CaO-Al_2_O_3_-Fe_2_O_3_-5wt%SiO_2_-2wt%MgO system calculated by FactSage 8.2. As shown in Fig. 6, according to the chemical composition of the pellets powder, the approximate location area of the rings is marked in the phase diagram^22,26^. With the increase of basicity(CaO/SiO_2_), the content of CaO increases. It is obvious that the position of the ring binding phase of magnesian fluxed pellets changes in the direction of the arrow. For the samples produced by magnesian fluxed pellets, the region moves to the lower temperature part. Therefore, more liquid phase will be produced during the roasting of the agglomerates of magnesian fluxed pellets. In a word, when the basicity of magnesian flux pellets increased, the composition of the binding phase in the ring changed significantly.

The change of the composition of the binding phase leads to a high proportion of the liquid phase in the rings, which leads to the adhesion between the pellet powder particles during the production of magnesian fluxed pellets in the rotary kiln. Therefore, basicity plays an important role in the formation of rings. With the increase of basicity, the ring forms a higher liquid phase, which will lead to more serious ring formation. Therefore, it is necessary to control the basicity of magnesian fluxed pellets to reduce the ring formation.

### Mechanism of ring formation

**Figure 7:**
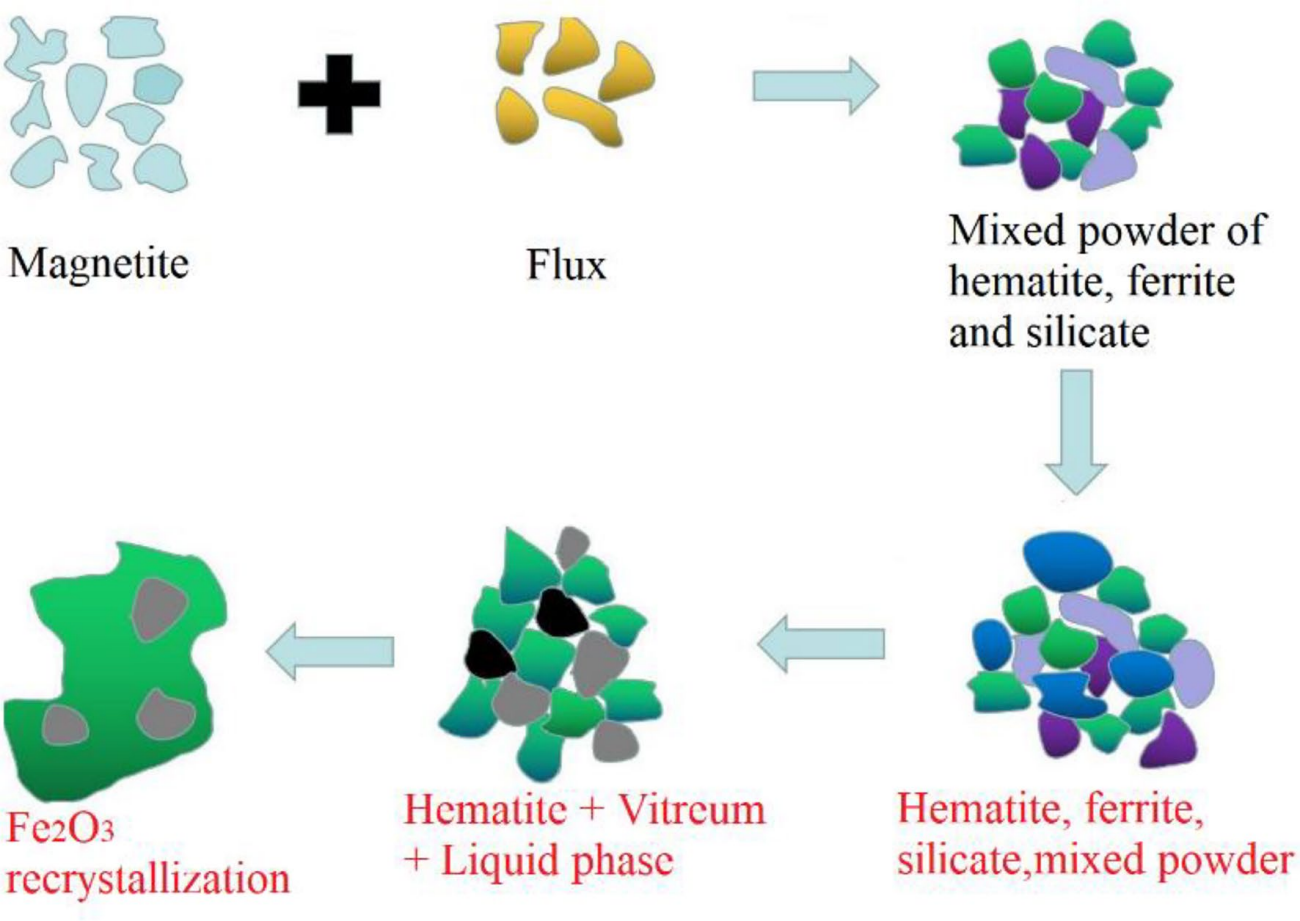
Ring formation mechanism of magnesian flux pellets in rotary kiln.

As shown in Fig. 7, during the ring formation process of magnesian flux pellet powder in the rotary kiln, magnetite was oxidized into hematite in the grate, with some hematite powder produced due to lower compressive strength of magnesian flux pellets. Pure hematite powder was difficult to form initial ring with high strength at 1250 °C. However, existence of CaO and MgO fluxes made hematite powder and fluxes able to produce liquid phases in the high-temperature rotary kiln, which promoted the diffusion and recrystallization of hematite in the rotary kiln, thus, improved the strength of initial ring and aggravated the ring formation in rotary kiln.

## Conclusions

1. With the increase of basicity, the briquette compressive strength of magnesian flux pellet powder increased gradually, due to which, rings were formed easily and it is difficult for briquettes of acid pellet powder to form rings for its lower compressive strength. Furthermore, under the condition of ensuring the pellet strength, the roasting temperature should be controlled below 1200 °C, which is a necessary condition for magnesian flux pellet powder to form the initial ring which was easy to be destroyed.
2. The briquette of magnesian flux pellet powder was mainly composed of hematite, magnetite, calcium ferrite, magnesium ferrite and olivine phases. With the increase of basicity, ferrite phase and olivine phase gradually increased, and iron olivine phase gradually became calcium iron olivine phase with lower melting point.
3. The aggravation of rings in rotary kiln was mainly due to the formation of liquid phase of magnesian flux pellet powder. In the rotary kiln, there were CaO and MgO fluxes produced by magnesian flux pellets, causing hematite powder and fluxes to produce liquid phases in the high-temperature rotary kiln, which promoted the diffusion and continuous crystallization of hematite, thus, improved the strength of the initial ring, and then intensified the ring formation in the rotary kiln.

## Data Availability

The datasets used and/or analyzed during the current study available from the corresponding author on reasonable request.

## References

[1] Wang RQ, Jiang L, Wang YD (2020). Energy saving technologies and mass-thermal network optimization for decarbonized iron and steel industry: A review. J. Clean. Prod..

[2] Guo Y, Liu K, Chen F (2021). Effect of basicity on the reduction swelling behavior and mechanism of limestone fluxed iron ore pellets. Powder Technol..

[3] Mohanty MK, Mishra S, Mishra B (2018). Effect of basicity on the reduction behavior of iron ore pellets. Arab. J. Sci. Eng..

[4] Iljana M, Kemppainen A, Paananen T (2015). Effect of adding limestone on the metallurgical properties of iron ore pellets. Int. J. Miner. Process..

[5] Lv W, Sun Z, Su Z (2019). Life cycle energy consumption and greenhouse gas emissions of iron pelletizing process in China, a case study. J. Clean. Prod..

[6] Bai K, Liu L, Pan Y (2021). A review: research progress of flux pellets and their application in China. Ironmak. Steelmak..

[7] Feng JX, Zhang Y, Zheng HW (2010). Drying and preheating processes of iron ore pellets in a traveling grate. Int. J. Miner. Metall. Mater..

[8] Sahu SN, Biswal SK (2021). Alleviating dependency on fossil fuel by using cow-dung during iron ore pelletization: Assessment of pellet physical and metallurgical properties. Powder Technol..

[9] Zhou F, Chen C, Zhou Pu (2021). The coupling effect in drying section in traveling grate: A CFD and experimental study. Min. Metall. Explor..

[10] Wang S, Guo Y, Liu K (2021). The deposit formation mechanism in coal-fired rotary kiln for iron ore pellet production: A review. Crystals.

[11] Bing Hu, Peiwei Hu, Biao Lu (2020). NOx emission reduction by advanced reburning in grate-rotary kiln for the iron ore pelletizing production. Processes.

[12] Jonsson CYC, Stjernberg J, Wiinikka H (2013). Deposit formation in a grate-kiln plant for iron-ore pellet production. Part 1: Characterization of process gas particles. Energy Fuels.

[13] Stjernberg J, Jonsson CYC, Wiinikka H (2013). Deposit, formation in a grate-kiln plant for iron-ore pellet production. Part 2: Characterization of deposits. Energy Fuels.

[14] Guo Y, Wang S, He Y (2017). Deposit formation mechanisms in a pulverized coal fired grate for hematite pellet production. Fuel Process. Technol..

[15] Sefidari H, Wiinikka H, Lindblom B, Nordin LO, Wu G, Yazhenskikh E, Müller M, Ma C, Öhman M (2019). Comparison of high-rank coals with respect to slagging/deposition tendency at the transfer-chute of iron-ore pelletizing grate-kiln plants: A pilot-scale experimental study accompanied by thermochemical equilibrium modeling and viscosity estimations. Fuel Process. Technol..

[16] Sefidari H, Lindblom B, Wiinikka H, Nordin L, Lennartsson A, Mouzon J, Bhuiyan IU, Öhman M (2008). The effect of disintegrated iron-ore pellet dust on deposit formation in a pilot-scale pulverized coal combustion furnace. Part II: thermochemical equilibrium calculations and viscosity estimations. Fuel Process. Technol..

[17] Stjernberg J, Jonsson CY, Wiinikka H, Lindblom B, Boström D, Öhman M (2013). Deposit formation in a grate–kiln plant for iron-ore pellet . Part II: Characterization of deposits. Energy Fuel.

[18] Wang S, Guo YF, Fan JJ (2018). Initial stage of deposit formation process in a coal fired grate-rotary kiln for iron ore pellet production. Fuel Process. Technol..

[19] Wang S, Guo YF, Fan JJ (2018). Deposits in a coal fired grate-kiln plant for hematite pellet production: Characterization and primary formation mechanisms. Powder Technol..

[20] Sefidari H, Ma C, Fredriksson C (2020). The effect of co-firing coal and woody biomass upon the slagging/deposition tendency in iron-ore pelletizing grate-kiln plants. Fuel Process. Technol..

[21] Wang S, Guo YF, Chen F, He Y, Jiang T, Zheng FQ (2016). Combustion reaction of pulverized coal on the deposit formation in the kiln for iron ore pellet production. Energy Fuel.

[22] Bale C, Bélisle E, Chartrand P, Decterov S, Eriksson G, Gheribi A, Hack K, Jung IH, Kang YB, Melançon J (2016). FactSage thermochemical software and databases 2010–2016. Calphad.

[23] Long HM, Chun TJ, Wang P, Meng QM, Di ZX, Li JX (2016). Grinding kinetics of vanadium-titanium magnetite concentrate in a damp mill and its properties. Metall Mater. Trans. B Process Metall. Mater. Process. Sci..

[24] Van Dyk JC, Benson SA, Laumb ML, Waanders B (2009). Coal and coal ash characteristics to understand mineral transformations and slag formation. Fuel.

[25] China Coal Industry Association (2008). GB/T 219-2008, Determination Method of Coal Ash Fusibility.

[26] Bale C, Bélisle E, Chartrand P, Decterov S, Eriksson G, Gheribi A, Hack K, Jung IH, Kang YB, Melançon J (2016). FactSage thermochemical software and databases, 2010–2016. Calphad.

[27] Lee WE, Souza GP, McConville GJ, Tarvornpanich T, Iqbal Y (2008). Mullite formation in clays and clay-derived vitreous ceramics. J. Eur. Ceram. Soc..

[28] Rezaie H, Rainforth WM, Lee WE (1997). Mullite evolution in ceramics derived from kaolinite, kaolinite with added α-alumina and sol–gel precursors. Br. Ceram. Trans..

[29] Tarvornpanich T, Souza GP, Lee WE (2005). Microstructural evolution on firing sodalime-silica glass fluxed whitewares. J. Am. Ceram. Soc..

[30] Zhong Q, Yang Y, Jiang T, Li Q, Xu B (2018). Effect of coal ash on ring behavior of iron-ore pellet powder in kiln. Powder Technol..

[31] Jiang, T., He, G. Q., Gan, M., Li, G. H., Fan, X. H. & Yuan, L. S. in (ed ICSTI), *Forming Mechanism of Rings in Rotary-Kiln for Oxidized Pellet, Proceedings of the 5th International Congress on the Science and Technology of Ironmaking*, Shanghai 292–297 (2009).

[32] Saxena SK, Chatterjee N, Fei Y, Shen G (1993). Thermodynamic Data on Oxides and Silicates.

[33] Yang, X. F. Fundamental and Applied Studies on Preparing Oxidized Pellets From Mixed Ore Concentrates. Ph.D. Thesis Central South University, China (2011) **(in Chinese)**.

[34] Jonsson CY, Stjernberg J, Wiinikka H, Lindblom B, Boström D, Öhman M (2013). Deposit formation in a grate-kiln plant for iron-ore pellet production. Part I: characterization of process gas particles. Energy Fuel.

