# Author Correction: Research on the ring formation mechanism of magnesian flux pellets in rotary kiln

**DOI:** 10.1038/s41598-023-30320-9

**Published:** 2023-02-28

**Authors:** Zongheng Guo, Tielei Tian, Yuzhu Zhang

**Affiliations:** College of Metallurgy and Energy, North China University of Science and Technology, Tangshan City, Hebei Province China

Correction to: *Scientific Reports*, 10.1038/s41598-023-29440-z, published online 10 February 2023

The Funding section in the original version of this Article was omitted. The Funding section now reads:

“This research was funded by the NATIONAL NATURAL SCIENCE FOUNDATION OF CHINA, Grant Number U20A20271; NATURAL SCIENCE FOUNDATION OF HEBEI PROVINCE, Grant Number E2020209184; SCIENCE AND TECHNOLOGY PROJECT OF TANG-SHAN CITY, Grant Number 20150217C; SCIENCE AND TECHNOLOGY PROJECT OF HEBEI EDUCATION DEPARTMENT, Grant Number ZD2021084.”

The original Article has been corrected.

